# Predicting Malignancy of Breast Imaging Findings Using Quantitative Analysis of Contrast-Enhanced Mammography (CEM)

**DOI:** 10.3390/diagnostics13061129

**Published:** 2023-03-16

**Authors:** Matthew M. Miller, Abu Hasnat Mohammad Rubaiyat, Gustavo K. Rohde

**Affiliations:** 1 Department of Radiology and Medical Imaging, University of Virginia Health System, 1215 Lee St., Charlottesville, VA 22903, USA; 2 Department of Electrical and Computer Engineering, University of Virginia, 415 Lane Rd., Charlottesville, VA 22903, USA; 3 Department of Biomedical Engineering, University of Virginia, 415 Lane Rd., Charlottesville, VA 22903, USA

**Keywords:** mammography, radiographic image enhancement, contrast media, computer-assisted image processing

## Abstract

We sought to develop new quantitative approaches to characterize the spatial distribution of mammographic density and contrast enhancement of suspicious contrast-enhanced mammography (CEM) findings to improve malignant vs. benign classifications of breast lesions. We retrospectively analyzed all breast lesions that underwent CEM imaging and tissue sampling at our institution from 2014–2020 in this IRB-approved study. A penalized linear discriminant analysis was used to classify lesions based on the averaged histograms of radial distributions of mammographic density and contrast enhancement. T-tests were used to compare the classification accuracies of density, contrast, and concatenated density and contrast histograms. Logistic regression and AUC-ROC analyses were used to assess if adding demographic and clinical data improved the model accuracy. A total of 159 suspicious findings were evaluated. Density histograms were more accurate in classifying lesions as malignant or benign than a random classifier (62.37% vs. 48%; *p* < 0.001), but the concatenated density and contrast histograms demonstrated a higher accuracy (71.25%; *p* < 0.001) than the density histograms alone. Including the demographic and clinical data in our models led to a higher AUC-ROC than concatenated density and contrast images (0.81 vs. 0.70; *p* < 0.001). In the classification of invasive vs. non-invasive malignancy, the concatenated density and contrast histograms demonstrated no significant improvement in accuracy over the density histograms alone (77.63% vs. 78.59%; *p* = 0.504). Our findings suggest that quantitative differences in the radial distribution of mammographic density could be used to discriminate malignant from benign breast findings; however, classification accuracy was significantly improved with the addition of contrast-enhanced imaging data from CEM. Adding patient demographic and clinical information further improved the classification accuracy.

## 1. Introduction

Mammographic screening has been shown to reduce breast cancer mortality [1,2,3], but concerns have arisen about whether mammography leads to too many benign biopsies or to a too frequent diagnosis of breast cancer in women for whom it would not have become clinically apparent in the patient’s lifetime with usual care, which is sometimes termed “overdiagnosis”. The benign biopsy rate would be reduced if the specificity of breast imaging could be improved, whilst overdiagnosis would be reduced if imaging features that better distinguish between clinically relevant and non-clinically relevant breast disease could be identified.

To help address the concerns of benign biopsies and overdiagnosis, quantitative analytic techniques have been applied to various breast imaging modalities in an attempt to discover new imaging features that better predict tumor behavior or patient outcomes. One common approach to the quantitative analysis of imaging data utilizes the automated extraction of imaging features from radiologist-delineated regions of interest (ROIs) followed by an evaluation for an association between these imaging features and histopathologic features. Several studies using this analytic approach have reported an association between automatically extracted breast magnetic resonance imaging (MRI) features and the malignancy or benignity of a finding [4,5,6,7]. Other similar studies have reported an association between the MRI features of malignant findings and the molecular subtype [8,9,10,11,12,13,14,15], the tumor grade [16], metastatic disease in the axillary lymph nodes [17], and disease recurrence [18].

Contrast-enhanced mammography (CEM) is an emerging imaging modality which, as with MRI, provides physiologic information derived from the use of intravenous contrast, which causes the enhancement of tissues that have increased blood flow, including many malignancies. CEM has been found by several studies to be both more sensitive and more specific than traditional mammography [19,20,21,22,23]. The high specificity of CEM makes it a promising imaging tool for reducing benign biopsies, whilst the physiologic component of CEM suggests that it could help reduce overdiagnosis through improved discrimination between clinically distinct types of breast disease. Compared with MRI and mammography, however, relatively little has been published so far exploring the diagnostic and prognostic utility of applying quantitative analytic techniques to CEM in the evaluation of suspicious breast findings.

The few early quantitative studies that have been performed using CEM have focused primarily on investigating the associations between automatically extracted CEM features and lesion malignancy or benignity [24,25,26,27,28,29], and between CEM features and the molecular subtype of invasive disease [30]. Most of these studies have reported promising results utilizing machine learning [24,26,27,28] or deep learning [25,29] techniques to predict tumor pathology features from CEM imaging features; however, these techniques are often difficult to interpret biologically or morphologically, and thus the underlying causes of imaging features often remain unclear.

In order to better understand the morphologic and biologic differences between malignant and benign breast findings, we sought to test whether simple and interpretable quantitative approaches to characterizing the spatial distribution of mammographic density and contrast enhancement within and around suspicious CEM findings could yield differences in diagnostic ability. We also sought to combine quantitative descriptions of contrast distribution with clinical and demographic patient information to create statistical models to predict malignancy vs. benignity, with the goal of facilitating a more accurate diagnostic and prognostic characterization of breast imaging findings and a more personalized and effective approach to patient care.

In this paper, we describe our exploration of different approaches to processing CEM imaging. We report that quantitative differences in the radial distribution of density on mammograms can discriminate between benign and malignant breast findings and that classification accuracy is significantly improved with the addition of contrast distribution data from CEM.

## 2. Materials and Methods

This retrospective study was Institutional Review Board-approved and compliant with the Health Insurance Portability and Accountability Act (HIPAA).

All patients who had undergone CEM imaging at our institution (2014–2020) were identified using our breast imaging tracking and reporting system, MagView (MagView, Fulton, MD, USA). Patients who did not go on to receive a biopsy or surgical excision of the suspicious finding were excluded from the study. The clinical radiology reports were reviewed for each patient and the following data were extracted: breast density; background parenchymal enhancement; number, type, and characteristics of the findings reported on each exam; associated BI-RADS designation for each individual finding; and whether each finding was biopsied and/or surgically excised. Patient demographic and clinical data, including age, race, past medical history of breast cancer, menopausal status, and breast cancer risk (Tyrer-Cuzick lifetime risk score), were extracted from the medical records. Pathologic data were extracted from the clinical pathology reports for the core needle biopsy and/or surgical excision for each suspicious lesion. If the biopsy pathology and surgical pathology differed, the worse diagnosis was used as the definitive pathology. Radiologic–pathologic concordance between the imaging and pathology findings were extracted from the radiology report addenda.

**Figure 1:**
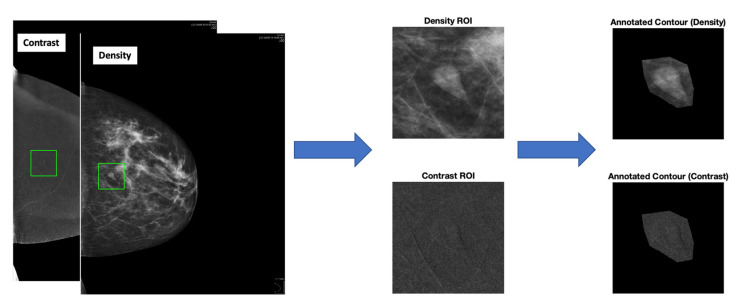
Process of image review and annotation. Each suspicious CEM lesion was annotated with a rectangular box ROI (green box) as well as a contoured, hand-drawn ROI in preparation for quantitative image analysis. The sizes of the mammogram images varied from patient to patient; however, the ROIs were chosen to be of a size 300 × 300 in pixels.

**Figure 2:**
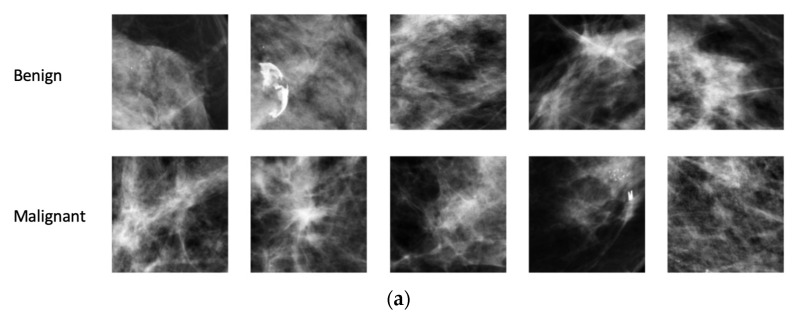

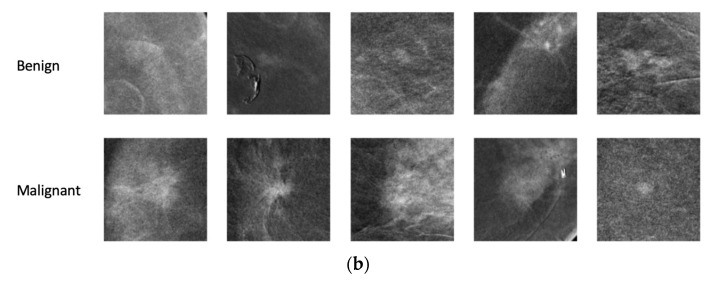
Sample ROIs extracted from (**a**) density and (**b**) contrast images.

The CEM images were annotated by a fellowship-trained breast imaging radiologist with four years of experience who was blinded to the pathology of the biopsied lesion. The images were reviewed and the regions of interest (ROIs) were drawn using a graphical user interface (GUI) using the clinical radiology reports and saved clinical markings on the images to guide annotation. This generally meant transferring the clinically drawn markings to the GUI and verifying the biopsy marker clip location on post-biopsy images to ensure that the intended target had been adequately biopsied. The images were annotated by drawing both a rectangular box ROI as well as a contoured, hand-drawn ROI around each finding using the GUI to prepare the image for the computational analysis (Figure 1 and Figure 2).

We explored several commonly used methodologies to determine the optimal method of analyzing our imaging data, including three neural network models ((1) a shallow convolutional neural network (CNN) model [31]; (2) a standard Resnet18 model [32]; and (3) a standard VGG11 model [33]); the Radon cumulative distribution transform (RCDT) [34], a transport-based image transformation technique used in combination with the nearest subspace classifier [31]; RCDT used in combination with a penalized linear discriminant analysis (PLDA); a PLDA of the gradient RCDT [35]; radial histogram PLDA; and radial histogram cumulative distribution transform (CDT)-PLDA (Supplementary Tables S1 and S2). The best accuracies for both density and contrast image analyses as well as the most physiologically interpretable results were seen with radial histogram PLDA, so the main portion of this paper focuses on describing these findings. The Supplementary Materials section shows the complete results, comparing the performance of all methods tried.

**Figure 3:**
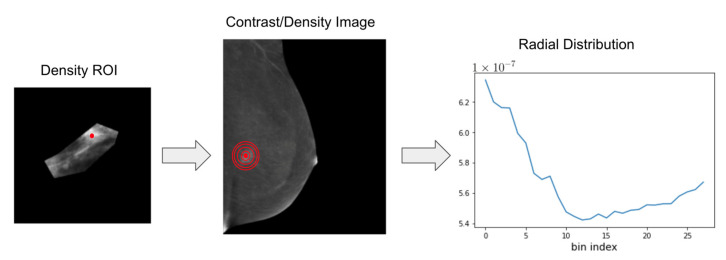
Creating histograms of radial distribution of density and contrast. Concentric bands (red circles) were automatically defined around the center of mass (red dot) of each finding and intensity values from each band were summed and normalized to create a histogram representing the distribution of density or contrast as a function of distance from the center of mass. The rightmost plot shows the histogram corresponding with the mammogram shown in the middle. Here, the x-axis represents the bin indexes corresponding with the concentric bands and the y-axis represents the normalized total mass (contrast/density) present in the bands.

To quantify the radial distribution of density (on the traditional mammogram) and contrast (on the contrast mammogram), we calculated the center of mass for each finding using the contoured ROI on the density image. Concentric circles were automatically defined around the center of mass, as quantified by the pixel intensity, on both the density and contrast images, creating a series of bands around the center of mass with an increasing distance from the center. The intensity values from each band were summed and normalized in accordance with the band’s area, and a histogram was created for each finding representing the distribution of density or contrast as a function of the distance from the center of mass (Figure 3). Average histograms were generated for all benign and all malignant findings as well as for all invasive and all non-invasive malignancies in preparation for the subsequent analysis. We also concatenated the density and contrast histograms for each finding subtype to ascertain the utility of their combined use.

PLDA was applied to the radial distributions [36]. Fisher’s linear discriminant analysis (LDA) is a commonly used tool for data analysis. PLDA is a penalized version of LDA, which is designed for situations where there are many highly correlated predictors. We divided the findings into benign findings (negative class) and malignant findings (positive class) for the primary set of experiments, and the malignancies were further subdivided into non-invasive malignancies and invasive malignancies for the secondary set of experiments. High-risk findings were excluded from these analyses. We used a 5-fold cross-validation with 5 repetitions. T-tests were used to compare the classification accuracies of the density, contrast, and concatenated density and contrast histograms.

We used logistic regression to create statistical models to predict the malignancy vs. benignity of the CEM findings using the clinical and demographic patient information combined with the quantitative descriptions of contrast and density distributions. This modeling included the variables of age, race, past medical history of breast cancer, menopausal status, and breast density. The performance of the predictive models was assessed by comparing the area under the curve for the receiver operating characteristic curve (AUC-ROC) for each model.

The data analysis was performed using Python Jupyter notebook (version: 6.0.3) and the lesion annotation was undertaken in MATLAB (version R2020a).

## 3. Results

### 3.1. Demographic, Imaging, and Pathology Data

During the study period, a total of 137 patients had at least 1 suspicious finding on CEM that subsequently underwent biopsy or surgical excision, with a total of 159 suspicious findings identified and sampled. The suspicious findings demonstrated a median patient age of 56.8 years (inter-quartile range (IQR): (47.7, 63.2)) (Table 1). Self-identified race was found to be 78.6% White, 14.5% Black, 2.5% Asian or Pacific Islander, and 4.4% Other Race or Race Not Recorded. A prior personal history of breast cancer was recorded for 37.7% of patients. Of those with no prior personal history of breast cancer, 35.4% were identified as high risk for breast cancer due to a Tyrer-Cuzick lifetime risk score of ≥20%. Medical records showed that 59.1% of patients were post-menopausal.

The suspicious findings reported on CEM included masses (40.3%; 64/159), asymmetries (28.3%; 45/159), calcifications (20.1%; 32/159), architectural distortion (5.7%; 9/159), and non-mass enhancement (5.0%; 8/159) (Table 1). The findings demonstrated a range of pathologies, with 44.0% (70/159) benign lesions, 6.3% (10/159) high-risk lesions, 5.0% (8/159) atypical lesions, and 44.7% (71/159) malignant lesions. An additional breakdown of the pathologies is listed in Table 1.

### 3.2. Malignant vs. Benign Classification Using Radial Distribution of Density and Contrast

**Figure 4:**
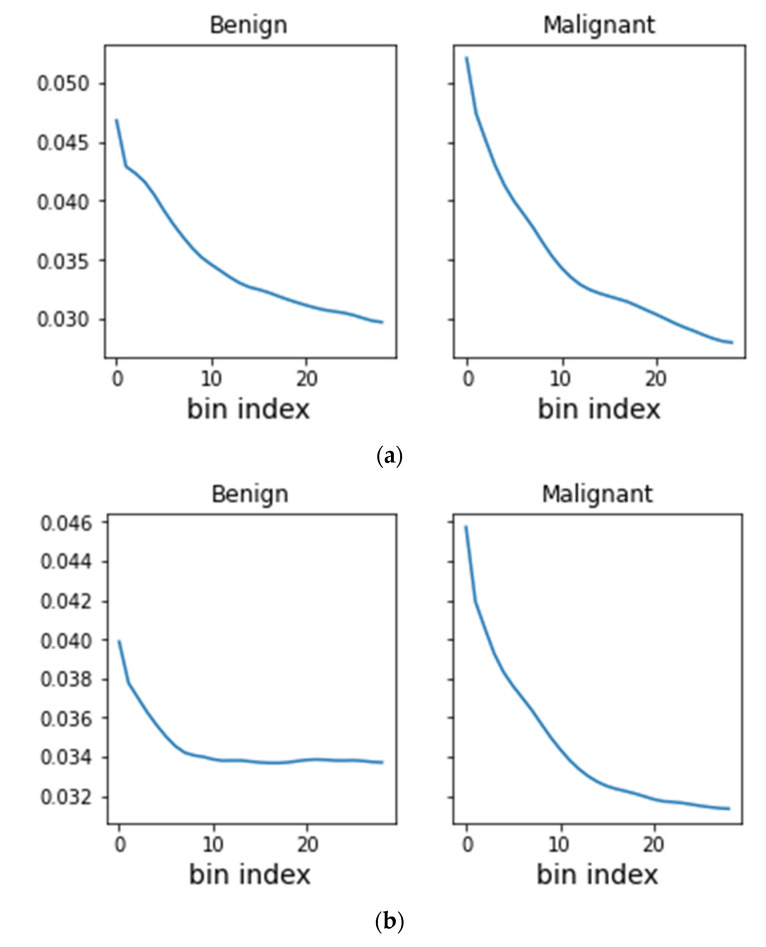
Mean radiographic and contrast density of benign and malignant findings as a function of distance from the calculated center of mass. Averaging across all CEM findings on (**a**) density images and (**b**) contrast images, malignant lesions demonstrated a higher concentration of mammographic density and intravenous contrast near the center of mass than benign lesions.

**Figure 5:**
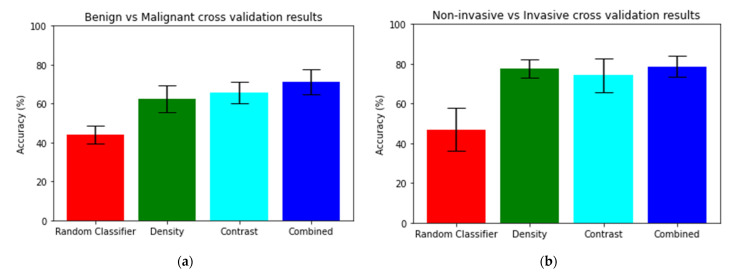
Accuracies of (**a**) malignancy vs. benignity classification and (**b**) invasive vs. non-invasive malignancy classification using radial histogram PLDA.

Malignant lesions were found to have a higher concentration of both mammographic density and intravenous contrast near the center of mass than the benign lesions (Figure 4). The PLDA of the averaged density and contrast radial histograms for the benign and malignant findings demonstrated that both the density and contrast histograms had a statistically significant (*p* < 0.001 and *p* < 0.001, respectively) higher classification accuracy than a random classifier, which had an accuracy of about 48% (Table 2 and Figure 5). There was no statistically significant difference between the classification accuracy of the density and contrast histograms, with the density histograms demonstrating an accuracy of 62.37% and the contrast histograms demonstrating an accuracy of 65.62% (*p* = 0.074). However, the analysis of the concatenated density and contrast histograms demonstrated a classification accuracy of 71.25%, which was a statistically significant improvement over both density alone (*p* < 0.001) and contrast alone (*p* = 0.002).

### 3.3. Statistical Modeling Using Imaging, Clinical, and Demographic Data

The univariable logistic regression analysis demonstrated that malignancy was associated with an older patient age (OR 1.07; 95% CI: [1.04, 1.10]), post-menopausal status (OR 3.81; 95% CI: [1.91, 7.58]), and non-dense breast tissue (OR 2.00; 95% CI: [1.05, 3.70]) (Table 3). On the multivariable analysis, the only factor independently associated with malignancy was an older patient age (AOR 1.05; 95% CI: [1.00, 1.10]).

**Figure 6:**
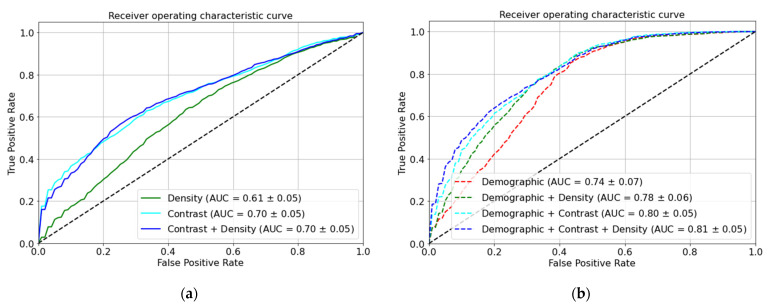
Malignant vs. benign ROC and AUC (**a**) without and (**b**) with demographic and clinical information (age, race, past medical history of breast cancer, menopausal status, and breast density). Inclusion of demographic and clinical data achieved a significantly higher AUC-ROC than imaging features alone. The black dotted line corresponds to the chance accuracy (AUC = 0.5).

Including the demographic and clinical data (age, race, past medical history of cancer, menopausal status, and breast density) in our models led to a significantly higher AUC-ROC than the density images alone (0.78 vs. 0.61; *p* < 0.001), contrast images alone (0.80 vs. 0.70; *p* < 0.001), or concatenated density and contrast images (0.81 vs. 0.70; *p* < 0.001) (Table 4 and Figure 6).

### 3.4. Non-Invasive vs. Invasive Malignancy Classification Using Radial Distribution of Density and Contrast

The PLDA of the averaged density and contrast radial histograms for non-invasive malignancies and invasive malignancies demonstrated that both the density and contrast histograms had a statistically significant (*p* < 0.001 and *p* < 0.001, respectively) greater accuracy than a random classifier, which had an accuracy of 47% (Table 2 and Figure 5). No statistically significant difference between the classification accuracy of the density and contrast histograms was seen, with the density histograms demonstrating an accuracy of 77.63% and the contrast histograms demonstrating an accuracy of 74.27% (*p* = 0.096). The analysis of the concatenated density and contrast histograms demonstrated a classification accuracy of 78.59% (*p* < 0.001), which was a statistically significant improvement over contrast alone (*p* = 0.0412) but not over density alone (*p* = 0.5040).

## 4. Discussion

We investigated new quantitative approaches of characterizing the spatial distribution of mammographic density and contrast enhancement within and around suspicious CEM findings identified by radiologists in order to better understand the nature of contrast enhancement in breast cancer and the utility of CEM in diagnosing and prognosticating breast cancer. Interestingly, among the several commonly used methodologies we explored, the simplest analysis—radial histogram PLDA—provided the most accurate and the most physiologically interpretable results with both density and contrast images.

For our primary classification task of classifying lesions as malignant or benign, we found that both the density and contrast radial histogram PLDA demonstrated a significantly higher classification accuracy than a random classifier, but a better classification accuracy was seen with the combined density and contrast radial histograms. These findings suggest that the contrast component of a CEM examination provides additional information not supplied by a traditional mammogram that can help with malignant vs. benign lesion classifications.

We also found that including demographic and clinical data into our CEM-based model led to a significantly higher AUC-ROC than using density images alone, contrast images alone, or a combination of both density and contrast images. In fact, the overall best performance in classifying lesions as malignant or benign was seen with the model combining concatenated density and contrast radial histograms with the demographic and clinical data (AUC-ROC 0.81). As our regression analyses found an older patient age to be the only tested demographic or clinical variable that was independently associated with lesion malignancy, adding patient age to the model based on CEM imaging features may be sufficient to obtain the best malignant/benign classification performance.

For our secondary classification task of classifying malignant lesions as invasive or non-invasive, we found that both the density and contrast radial histogram PLDA had greater accuracy than a random classifier for predicting invasive vs. non-invasive malignancies. The concatenated density and contrast histograms demonstrated a statistically significant improvement over contrast alone but not over density alone. These findings suggest that for the classification of invasive vs. non-invasive malignancies, contrast images might not contain additional information not already included in the density images.

Our findings demonstrated that both mammographic density and contrast enhancement were distributed with a greater central concentration in breast malignancies than in benign findings, a fact that we found could be exploited to better discriminate malignant from benign findings. Similarly, we found that mammographic density was distributed with a greater central concentration with invasive malignancies than non-malignant malignancies. As these differences in radial distribution are often not readily apparent on a visual inspection, our findings indicated that there may be a role for computer-aided diagnosis in the interpretation of CEM examinations. The utility of this distribution of density and contrast needs to be validated on larger and prospective datasets, but could eventually play a role in helping the radiologist to better categorize a finding as benign or malignant, which would potentially help to decrease benign biopsies. Similarly, an analysis of the distribution of mammographic density might facilitate distinguishing invasive from non-invasive malignancies, which could help reduce overdiagnosis by helping to distinguish patients with DCIS who have occult invasive disease (and need surgical treatment) from those who do not (possibly manageable with active surveillance).

This study had a few limitations. This study was a single-institution, retrospective study; therefore, the results may not apply to other institutions or clinical settings. The patients included in this study were patients for whom a CEM was clinically indicated, most often performed as a cancer staging study or an MRI-directed biopsy planning study, so these cases might not be representative of cases encountered in other diagnostic or screening settings.

## 5. Conclusions

In conclusion, quantitative differences in the radial distribution of density on mammograms could be used to discriminate between benign and malignant breast findings; however, classification accuracy was significantly improved with the addition of contrast distribution data from CEM. The higher degree of a central concentration of density and contrast in malignant lesions compared with benign lesions was the discriminating feature in this classification task. Patient demographic and clinical information further improved the classification accuracy of the CEM-based models. Our findings help lay the groundwork for building models based on CEM features and clinical and demographic data to improve the diagnostic and prognostic characterization of imaging findings in the breast, which could eventually help to reduce benign biopsies and overdiagnosis, and facilitate a more personalized and effective approach to patient care.

## Figures and Tables

**Table 1:** Summary of demographic, imaging, and pathology data for all lesions detected on CEM (*n* = 159). IQR: inter-quartile range.

Demographic Data	
Age (Median (IQR))	56.8 (47.7, 63.2)
Race	
White	78.6% (125/159)
Black	14.5% (23/159)
Asian or Pacific Islander	2.5% (4/159)
Other Race or Race Not Recorded	4.4% (7/159)
Prior Personal History of Breast Cancer	
No	62.3% (99/159)
Not High Risk (<20%)	60.6% (60/99)
High Risk (≥20%)	35.4% (35/99)
Unknown	4.0% (4/99)
Yes	37.7% (60/159)
Menopausal Status	
Pre-Menopausal	40.9% (65/159)
Post-Menopausal	59.1% (94/159)
Imaging Data	
Breast Density	
Fatty	6.3% (10/159)
Scattered	43.4% (69/159)
Heterogeneously Dense	36.5% (58/159)
Extremely Dense	13.8% (22/159)
Background Parenchymal Enhancement	
Minimal	73.6% (117/159)
Moderate	23.2% (36/159)
Marked	3.9% (6/159)
Suspicious Finding Category	
Mass	40.3% (64/159)
Asymmetry	28.3% (45/159)
Calcifications	20.1% (32/159)
Architectural Distortion	5.7% (9/159)
Non-Mass Enhancement (NME)	5.0% (8/159)
Solitary Dilated Duct	0.6% (1/159)
Pathology Data	
Pathology	
Benign	70
Benign Fibrous Tissue/Fibrosis/Stromal Fibrosis	16
Fibrocystic	13
Fibroadenoma	7
Usual Ductal Hyperplasia (UDH)	6
Apocrine Metaplasia	4
Sclerosing Adenosis	4
Fat Necrosis	4
Benign Breast Parenchyma	4
Pseudoangiomatous Stromal Hyperplasia (PASH)	2
Mastitis	2
Lobular Atrophy	2
Other	6
High Risk	10
Papilloma	6
Radial Scar	4
Atypia	8
Atypical Ductal Hyperplasia (ADH)	4
Lobular Carcinoma In Situ (LCIS)	2
Atypical Lobular Hyperplasia (ALH)	1
Atypical Papilloma	1
Malignant	71
Invasive Ductal Carcinoma (IDC)	44
Ductal Carcinoma In Situ (DCIS)	19
Invasive Lobular Carcinoma (ILC)	5
Invasive Carcinoma, Not Otherwise Specified	1
Papillary Carcinoma In Situ	1
Tubular Carcinoma	1

**Table 2:** Classification performance of models utilizing radial histogram PLDA for density images, contrast images, and concatenated density and contrast images. W.r.t.: with respect to; * statistically significant (*p* ≤ 0.05).

	Random Classifier	Density	Contrast	Concatenated Density and Contrast
Classification Task: Malignant vs. Benign				
Accuracy	48%	62.37%	65.62%	71.25%
*p*-value (w.r.t. random classifier)		<0.001 *	<0.001 *	<0.001 *
*p*-value (w.r.t. density)		-	0.074	<0.001 *
*p*-value (w.r.t. contrast)		-	-	0.002 *
Sensitivity		0.6295	0.5616	0.6834
Specificity		0.6212	0.7591	0.7485
F1 score		0.6210	0.6087	0.6990
Classification Task: Invasive vs. Non-invasive Malignancy				
Accuracy	47%	77.63%	74.28%	78.59%
*p*-value (w.r.t. random classifier)		<0.001 *	<0.001 *	<0.001 *
*p*-value (w.r.t. density)		-	0.0963	0.5040
*p*-value (w.r.t. contrast)		-	-	0.0412 *
Sensitivity		0.7767	0.6918	0.7845
Specificity		0.7607	0.8677	0.7921
F1 score		0.8244	0.7888	0.8335
Kappa score		0.4673	0.4518	0.4988

**Table 3:** Association between predictor variables and malignancy. OR: odds ratio; AOR: adjusted odds ratio; All Other Races includes Black, Asian or Pacific Islander, Other Race, or Race Not Recorded; * statistically significant (*p* ≤ 0.05). The numbers in brackets are the 95% CI.

	Univariable	Multivariable
Predictor Variable	OR [CI]	AOR [CI]
Ratio		
Age		
	1.07 [1.04, 1.10] *	1.05 [1.00, 1.10] *
Race		
White	-	-
All Other Races	0.84 [0.39, 1.80]	0.73 [0.31, 1.70]
Personal History of Breast Cancer		
No	-	-
Yes	1.76 [0.92, 3.36]	1.69 [0.84, 3.42]
Menopausal Status		
Pre-Menopausal	-	-
Post-Menopausal	3.81 [1.91, 7.58] *	1.48 [0.48, 4.56]
Breast Density		
Non-dense	-	-
Dense	0.50 [0.27, 0.95] *	0.66 [0.32, 1.33]
Background Parenchymal Enhancement		
Minimal/Moderate	-	-
Marked	0.53 [0.25, 1.10]	0.76 [0.33, 1.75]

**Table 4:** Comparison of model accuracies with and without inclusion of demographic and clinical data (age, race, past medical history of breast cancer, menopausal status, and breast density). AUC-ROC: area under the curve for the receiver operating characteristic curve. * statistically significant (*p* ≤ 0.05).

	AUC-ROC Without Demographic and Clinical Data	AUC-ROC With Demographic and Clinical Data	*p*-Value
Density	0.61	0.78	<0.001 *
Contrast	0.70	0.80	<0.001 *
Concatenated Density and Contrast	0.70	0.81	<0.001 *
Without Imaging Data	-	0.74	-

## Data Availability

The data presented in this study are available on request from the corresponding author.

## Supplementary Materials

The following supporting information can be downloaded at https://www.mdpi.com/article/10.3390/diagnostics13061129/s1, Table S1: Malignant vs. benign classification performance of models utilizing various quantitative methods for analysis of density ROIs; Table S2: Malignant vs. benign classification performance of models utilizing various quantitative methods for analysis of contrast ROIs. References for supplementary materials: [31,32,33,34,35,36].
