# Supplementary material for: Predicting Malignancy of Breast Imaging Findings Using Quantitative Analysis of Contrast-Enhanced Mammography (CEM)
Source: Diagnostics (Basel). 2023 Mar 16;13(6):1129. doi: 10.3390/diagnostics13061129 (PMC10047016; doi:10.3390/diagnostics13061129)
Supplement: Supplementary file 1 [file diagnostics-13-01129-s001.zip › diagnostics-2250657-supplementary.pdf]

Supplemenatry Materials

Optimizing Methodology of Analysis

In addition to PLDA analysis of the normalized radial histograms of intensity values described in this paper, we also tested the application of several other methods to the same malignant vs. benign classification problem, including three neural network models: (1) a shallow convolutional neural network (CNN) model [31], (2) standard Resnet18 model [32], and (3) standard VGG11 model [33]. In addition, the Radon Cumulative Distribution Transform (RCDT) [34], a transport-based image transformation technique, was used in combination with nearest subspace classifier (RCDT-NS) [31] and penalized linear discriminant analysis (RCDT-PLDA) to classify the density and contrast mammograms. We also conducted a PLDA analysis of the gradient-RCDT [35] of the images to quantify the gradient distribution of density and contrast mammograms. PLDA analysis of the radial histogram-Cumulative Distribution Transform (CDT) was also performed. The classification results are summarized in Supplemental Tables 1 and 2.

Table S1. Malignant vs. benign classification performance of models utilizing various quantitative methods for analysis of density ROIs. CNN = convoluted neural network, RCDT = radon cumulative distribution transform, CDT = cumulative distribution transform.

Methods	Accuracy (%)	Sensitivity	Specificity	F1 score
ShallowCNN	54.04	0.6033	0.4813	0.5624
Resnet18	50.99	0.4063	0.6373	0.3865
VGG11	50.32	0.2360	0.8063	0.2262
RCDT-NS	54.65	0.6208	0.4781	0.5762
RCDT-PLDA	55.79	0.5707	0.5462	0.5588
Gradient-RCDT	61.48	0.6827	0.5504	0.6378
Radial-Histogram	62.37	0.6295	0.6212	0.6210
Radial-Histogram-CDT	59.87	0.6497	0.5550	0.6117

Table S2. Malignant vs. benign classification performance of models utilizing various quantitative methods for analysis of contrast ROIs. CNN = convoluted neural network, RCDT = radon cumulative distribution transform, CDT = cumulative distribution transform.

Methods	Accuracy (%)	Sensitivity	Specificity	F1 score
ShallowCNN	64.83	0.6343	0.6718	0.6378
Resnet18	52.95	0.3961	0.6942	0.3907
VGG11	57.26	0.4414	0.7133	0.44
RCDT-NS	59.24	0.7631	0.4252	0.6514
RCDT-PLDA	64.86	0.6734	0.6291	0.6571
Gradient-RCDT	49.25	0.4779	0.5098	0.4817

Radial-Histogram	65.62	0.5616	0.7591	0.6080
Radial-Histogram-CDT	64.50	0.5572	0.7413	0.5990
